# Strong Hydrogen Bonds Sustain Even–Odd Effects in Poly(ester amide)s with Long Alkyl Chain Length in the Backbone

**DOI:** 10.1021/acs.biomac.4c01191

**Published:** 2024-10-30

**Authors:** Leire Sangroniz, Jorge L. Olmedo-Martínez, Wenxian Hu, Yoon-Jung Jang, Guoming Liu, Marc A. Hillmyer, Alejandro J. Müller

**Affiliations:** 1 Department of Chemistry, 5635 University of Minnesota, Minneapolis 55455-0431, United States; 2 POLYMAT and Department of Polymers and Advanced Materials: Physics, Chemistry and Technology, Faculty of Chemistry, 160665 University of the Basque Country UPV/EHU, Paseo Manuel de Lardizábal, 3, Donostia-San Sebastián 20018, Spain; 3 Beijing National Laboratory for Molecular Sciences, CAS Key Laboratory of Engineering Plastics, Institute of Chemistry, 53030 Chinese Academy of Sciences, Beijing 100190, China; 4 University of Chinese Academy of Sciences, Beijing 100049, China; 5 IKERBASQUE, Basque Foundation for Science, Plaza Euskadi 5, Bilbao 48009, Spain

## Abstract

The number of methylene groups between strongly interacting functional groups within polymer repeating units induces even–odd effects on thermal and mechanical properties. However, detailed studies correlating the even–odd effect with structural changes are still lacking. In this work, we establish correlations between the structure and thermal properties of poly­(ester amide)­s containing long alkyl chain lengths. The even–odd effect impacts the thermal properties, including the melting temperature and crystallinity degree. It influences the spherulitic morphology of poly­(ester amide)­s, controlling the appearance of banding. We demonstrate that even–odd effects in poly­(ester amides)­s persist even with 27 CH_2_ groups within the repeating unit, an effect due to strong hydrogen bonds caused by the amide groups. Our X-ray studies reveal that the even–odd effect originates from changes in the crystalline structure of the materials. This work helps elucidate the role of strong intermolecular interactions (i.e., hydrogen bonding) on the even–odd effect in long-chain poly­(ester amides).

## Graphical abstract

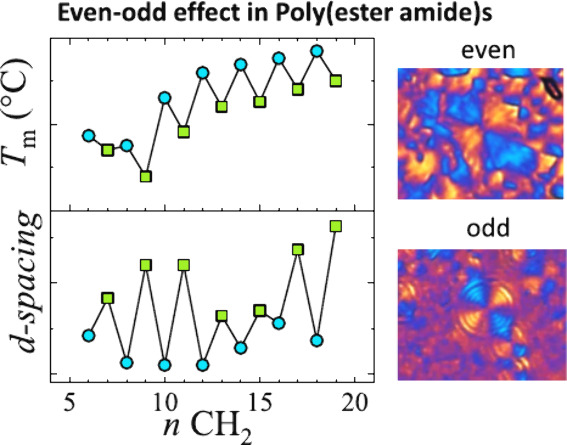

## 1 Introduction

Plastics are highly versatile materials with applications in countless areas, including packaging, construction, the automotive industry, and biomedicine, due to their advantages: polymers can be produced cost-effectively, exhibit low densities, and are easily processed. Since the end of life of plastic products has not been considered in commercial materials, the plastic waste that escapes the collection step is accumulated in the environment. Furthermore, nonrenewable sources are mainly used to produce commercial polymers. To overcome this issue, in the last decades, great effort has been made to develop sustainable polymers that can be obtained from renewable sources instead of petroleum and that can be degraded or chemically recycled.^1,2^

An interesting biobased and biodegradable polymer family includes poly­(ester amide)­s (PEA), which combine the degradable nature of polyesters (as the ester group can be hydrolytically cleaved) and the good thermal and mechanical properties of polyamides (originating from strong hydrogen bonds of amide groups). Many PEAs are semicrystalline, so their final performance depends on their crystallinity degree and melting points.^3,4^ In fact, in semicrystalline polymers, high crystallinity leads to higher biostability,^5^ Young’s modulus, and yield stress,^6^ and lower permeability to gases.^7,8^ Thus, it is of paramount relevance to understand the structure–property relationship to fine-tune the performance of the materials for specific applications.

The thermal properties of polymers depend on the chemical nature, thermal history, and presence of additives or other polymers.^9,10^ Efforts to understand the relationship between the chemical structure of polymers and their properties have been made over the past several decades. For example, the impact of the alkyl chain length of the main backbone on the thermal properties such as melting or crystallization temperature has been studied, revealing an alternating (zigzag) behavior with the number of CH_2_ in the main chain depending on whether the polymer has an even or an odd number of CH_2_ groups in its repeating unit.^11,12^ More recently, detailed studies of the crystallization kinetics have shown the same alternating trend.^13–16^ This effect is not limited to thermal properties; several works have reported a zigzag behavior on the mechanical properties of polyesters^17^ or polyurethane elastomers^18^ and on the optical properties of alkoxy side-chain substituted phenylene-ethynylene and phenylene-vinylene polymers.^19^ The alternating behavior of properties with methylene groups has been termed the even–odd effect if the properties such as melting or crystallization temperature are higher for polymers with an even number of CH_2_s than the materials with an odd number of CH_2_s. In contrast, the odd–even term is used in the opposite case.^14^

The even–odd effect has been reported for several polymers, such as polyesters, polyamides, and polyurethanes. In the past decade, a few studies have been carried out with polymer families containing a large number of CH_2_ groups in their repeating units, such as arylated polyesters,^20,21^ polyacetals,^22^ polycarbonates,^13^ and polyethers,^14^ revealing that it is possible to reach a saturation region in which the even–odd effect is no longer apparent. This is reflected in the thermal properties showing a monotonic trend with the number of CH_2_ beyond a critical number of methylene units. The saturation effect arises from dilution of the intermolecular interactions provided by the functional groups. Increasing the number of CH_2_ in the repeating unit, London dispersion forces are more relevant for the packing structure than dipole–dipole or hydrogen bond interactions arising from any functional groups present.^22^ On the contrary, saturation has not been observed for polymers such as polyurethanes^26^ or polyamides.^23–25^ In the case of polyamides with a PA XY structure, intermediate alkyl chain lengths were studied without reaching a saturation region. The even–odd effect arises from the differences in the packing chain structure within the crystal.^23–25^ The studies have been extended to aliphatic polyesters with up to 10 CH_2_ groups in the diol part, which also display an even–odd effect. The authors correlate this even–odd effect with the differences in the diffraction angle values.^17^ However, polymers with longer alkyl chain lengths in the main backbone have not been targeted to ascertain whether the even–odd effect is no longer apparent after reaching a saturation regime.

Although the even–odd effect of various systems has been reported in the literature, detailed studies investigating the structure–property relationship are still lacking, which would allow an understanding of the origin of this effect. Since amide groups introduce strong hydrogen bonds, poly­(ester amide)­s (PEAs) are good candidates for investigating the persistence of the even–odd effect when long (e.g., >12 CH_2_ units) alkyl chain lengths are included in the repeating unit. In a previous study, we reported the synthesis of PEAs derived from glycolide, a monomer that can be renewably sourced, with good mechanical performance and barrier properties, and that could be degraded under hydrolytic conditions.^27^ We have previously studied the impact of introducing functional groups into polyesters, addressing their crystallization kinetics^28^ and melt memory effect.^29^ However, the impact of the alkyl chain length in these materials has not been explored.^12,27,30–32^

Here, we investigate aliphatic PEAs with much longer alkyl chains than those previously reported in the literature, studying the correlation between the thermal properties and the crystalline structure, which reflects the chain packing of the polymers within the crystals. Employing PEAs with longer alkyl chains allows focusing on the role of intermolecular interactions by careful isolation of the interacting functional groups in contrast with noninteracting alkyl segments. We synthesized PEAs with eight CH_2_ groups in the diol part and varied the number of CH_2_ groups in the diacid part from 6 to 19 methylene groups. The originality of this work includes long alkyl chain lengths studied for the first time with samples of up to 27 CH_2_ groups in the repeating unit. Additionally, we have studied in detail the microstructure of the synthesized polymers correlating it with the alkyl chain length, which has not been addressed before for such kinds of systems. Thus, the role of intermolecular interactions on the even–odd effect has been determined, paving the way for the design of semicrystalline poly­(ester amide) systems with tailored properties.

## 2 Experimental Section

### 2.1 Materials

Poly­(ester amide)­s were synthesized following the procedure reported previously employing a diamidodiol and the appropriate diacid.^27^ A series of PEAs with varied numbers of methylene groups in the diacid from 6 to 19 were prepared. The diacids were purified by recrystallization with acetone and water before use. The chemical structures of the PEAs studied are given in Scheme 1. The molar mass and dispersity of the materials determined by size exclusion chromatography (SEC) are displayed in Table 1 (the SEC data and measurement details are provided in the Supporting Information). In the sample code, PEA8-*y*, the first number, 8, corresponds to the number of CH_2_ groups in the diamidodiol (constant for this study), and the second number, *y*, refers to the number of CH_2_ groups in the diacid part (6–19).

**Scheme 1:**
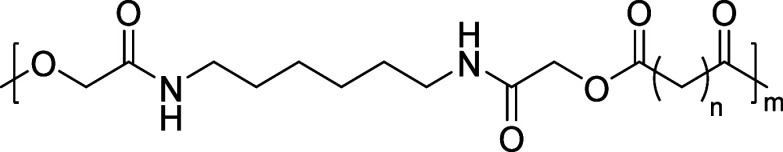
Chemical Structure of PEAs Studied in This Work^a^

a The number of methylene groups in the diacid part (*n* in the scheme) has been varied from 6 to 19.

**Table 1:** Sample Code, Number of Methylene Groups in the Diacid Part, Number Average Molar Mass, Weight Average Molar Mass, and Dispersity Obtained by SEC

sample	*n* CH_2_ diacid part	*M*_n_ (kg/mol)	*M*_w_ (kg/mol)	*Đ* (*M*_w_/*M*_n_)
PEA8-6	6	16.0	30.4	1.9
PEA8-7	7	16.0	28.8	1.8
PEA8-8	8	13.8	29.0	2.1
PEA8-9	9	14.1	31.0	2.2
PEA8-10	10	17.9	39.4	2.2
PEA8-11	11	15.0	31.5	2.1
PEA8-12	12	12.4	24.8	2.0
PEA8-13	13	11.9	31.0	2.6
PEA8-14	14	4.1	12.7	3.1
PEA8-15	15	4.1	14.8	3.6
PEA8-16	16	3.2	8.6	2.7
PEA8-17	17	4.2	14.3	3.4
PEA8-18	18	2.2	9.2	4.2
PEA8-19	19	2.7	9.7	3.6

Considering the molar mass, the samples can be divided into two groups. One group formed with PEAs with up to 13 methylene groups in the diacid part that have number average molar masses around 14 kg/mol, i.e., medium molar mass. The second group, PEAs with 14–19 methylene groups, have lower molar masses, around 4 kg/mol, i.e., low molar mass group. For the low molar mass samples, unexpectedly, a smooth trend was observed in the thermal properties with the number of CH_2_ groups. Thus, we assume in the study that both sets of polymers have sufficient molar mass to make fair comparisons among the series.

### 2.2 Differential Scanning Calorimetry (DSC)

The DSC experiments were carried out with a PerkinElmer DSC 8000 connected to an Intracooler II. The measurements were performed under an ultrapure nitrogen flow (20 mL/min). The instrument was previously calibrated with tin and indium. Samples were dried under a vacuum at 70 °C for 2 days before the experiments were performed, and a sample weight of about 5 mg was used for each experiment.

Nonisothermal experiments were carried out by first heating the material from room temperature to a high enough temperature to remove the thermal history, usually *T*_m,end_ + 25 °C. This initial heating scan is called the first heating scan. The sample was held in the melt for 5 min to remove the thermal history. Then, it was cooled down to 0 °C to acquire the cooling scan. Finally, it was heated again to the appropriate melting temperature, obtaining the second heating scan. The heating and cooling scans were performed at 20 °C/min.

The overall crystallization kinetics of the materials were investigated by performing isothermal experiments. For that, the recommendations of Müller et al. were followed.^33,34^ First, the minimum *T*_c_ was determined, i.e., the minimum temperature at which the sample cannot crystallize during the previous cooling step, at 60 °C/min. For that, the sample was heated to an appropriate temperature to remove thermal history, spending 5 min in the melt, and then it was cooled at 60 °C/min to the selected *T*_c_ temperature. When the *T*_c_ value was reached, the sample was immediately heated again. If a melting peak appears in the subsequent heating scan from *T*_c_, then it means that the sample crystallized during the previous cooling. The procedure was repeated, varying the *T*_c_: remove the thermal history, cool to selected *T*_c_ at 60 °C /min, and heat immediately. Once a *T*_c_ value at which the material crystallized during cooling was found, the *T*_c_ was increased in the following steps until it reached a *T*_c_ value for which there was no melting in the subsequent heating. Thus, the minimum *T*_c_ is the lowest temperature at which there is no melting peak in the subsequent heating. In this way, it is ensured that crystallization proceeds only isothermally (not during the cooling process) at the selected *T*_c_.

Once *T*_c,min_ was determined, isothermal tests were carried out by erasing the thermal history and cooling at 60 °C/min to the selected *T*_c_. At *T*_c_, enough time was spent to ensure that the crystallization process was completed. Then, the sample was heated at 20 °C/min to record the melting endotherm of the crystals formed during the isothermal process. The measurement started at *T*_c,min_, and the whole process was repeated by selecting higher *T*_c_ values than *T*_c,min_. In this case, a fresh sample was used for each *T*_c_ to avoid degradation.

### 2.3 Polarized Light Optical Microscopy

The microstructure of the samples was studied by employing an Olympus BX51 polarized light optical microscope, and the micrographs were acquired with an Olympus SC50 camera. The samples were melted at the appropriate temperature to remove the thermal history and cooled down at 20 °C/min, following the same procedure used in the nonisothermal experiments carried out in the DSC. This thermal procedure was applied with a Linkam THMS600 hot stage connected to a liquid nitrogen vessel to control the cooling.

### 2.4 Small-Angle X-ray Scattering (SAXS)

SAXS experiments were performed at the ESRF Synchrotron in Grenoble (France) at the BM26 beamline. The samples were thermally treated using the same thermal procedure as in the nonisothermal experiments carried out by DSC (heating and cooling from the melt at 20 °C/min). An X-ray source with a wavelength of 0.10332 nm was used at 12 keV. The diffraction signal was detected employing a Pilatus 1 M detector, with the distance between the sample and the detector being 2.95 m. The *q*-range was previously calibrated with silver behenate.

### 2.5 Wide Angle X-ray Scattering (WAXS)

WAXS patterns were acquired by employing a Xeuss 2.0 SAXS/WAXS system (Xenocs, France). The instrument produced a multilayer focused Cu Kα X-ray radiation beam and was equipped with a semiconductor detector (PILATUS 300K, DECTRIS, Switzerland). The PEA samples were cooled slowly (∼4 °C/min) from the isotropic melt state to room temperature to increase the crystallinity. Two sample-to-detector distances were applied to cover a broad scattering angle range (126.1 and 290.2 mm). After normalization by the overlapping region, the 1D WAXS profiles were joined from the two separate tests.

The studies as a function of temperature were carried out at the ESRF; the details of the X-ray source are given in Section 2.4. The previously thermally treated samples were heated inside aluminum DSC pans at 20 °C/min with a Linkam THMS600 hot stage. A Pilatus 300K-WF detector was used with a sample-to-detector distance of 279 mm.

## 3 Results and Discussion

### 3.1 Nonisothermal Crystallization

The thermal properties of poly­(ester amide)­s have been investigated by performing nonisothermal experiments. The cooling scans (Figure 1) indicate that all the materials studied can crystallize under the studied conditions (at 20 °C/min), showing a single exothermic peak. The crystallization peak is very small and broad for samples with six to nine methylene groups in the diacid part. PEA8-13 shows a high-temperature tail in the crystallization, which could result from the formation of different crystal forms (polymorphism) or the crystallization of chains with different lengths. During the subsequent heating scans, PEAs with a small number of methylene groups display a single melting peak, whereas above 12 methylene groups, complex melting curves with several endothermic peaks were observed. PEAs with six to nine CH_2_ groups show cold crystallization followed by a melting peak, since during the previous cooling scan, materials were not allowed to crystallize until saturation at the selected rate. The complex melting behavior of PEAs with long alkyl chain lengths could arise from polymorphism or crystal reorganization processes.

**Figure 1:**
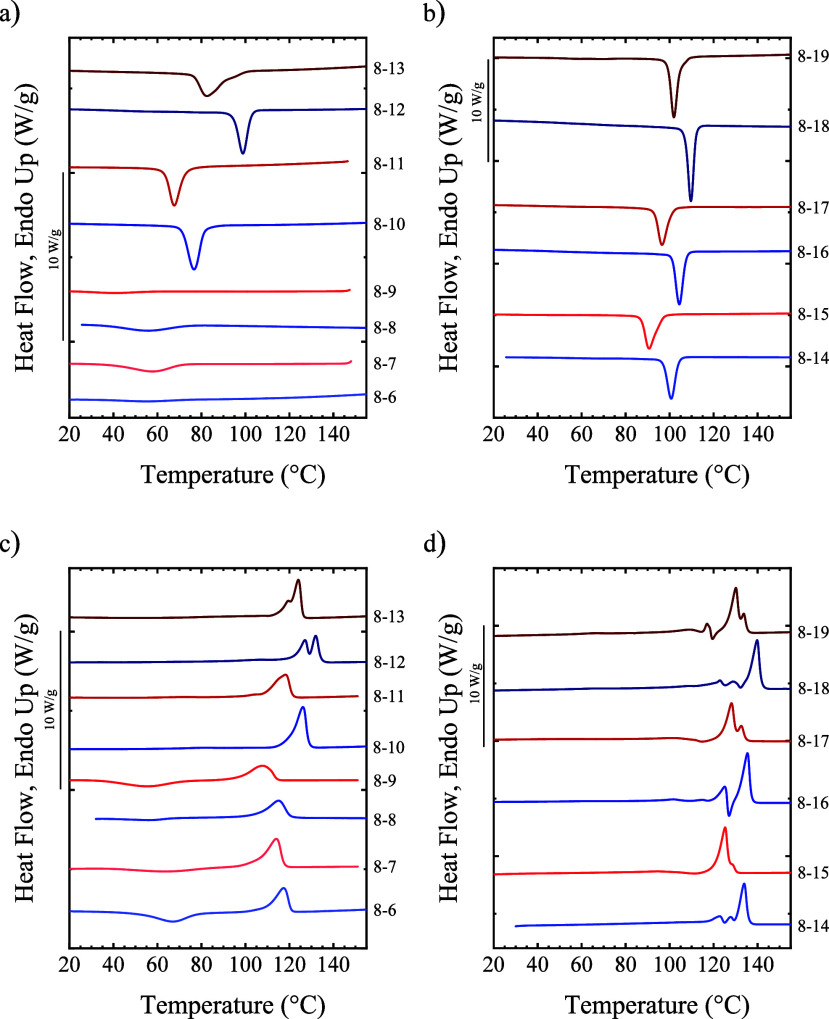
Cooling DSC scans from the melt for (a) PEAs with 6–13 CH_2_ groups in the diacid part and (b) 14–19 CH_2_ groups. Subsequent heating scans at 20 °C/min for (c) PEAs with 6–13 CH_2_ groups in the diacid part and (d) 14–19 CH_2_ groups.

To distinguish these possibilities, X-ray experiments were carried out as a function of the temperature. Complex diffraction patterns of the materials with several overlapping peaks were observed. In the cases of PEA samples with 12, 14, 16, 18, and 19 CH_2_ groups in the diacid part, different diffraction peaks were observed when heating, suggesting that several polymorphs are formed (see Figure S2). However, reorganization cannot be completely ruled out. The behavior of PEA samples with 15 and 17 CH_2_ groups is not clear since only a small diffraction peak is displayed and disappears at low temperatures, so reorganization of the crystals is probably occurring (see Figure S2). A reorganization process of the crystalline phase during heating has been reported for several polymer families, including poly­(ester amide)­s.^28,32,35,36^

Both crystallization and main melting peaks increase (alternatively) with the number of methylene groups (Figure 2a and 2b; for samples containing several peaks, the most intense was selected). Considering that high-density polyethylene has a melting temperature of 130 °C and the PEAs have lower *T*_m_ values, it could be expected that the introduction of methylene groups increases *T*_m_ since the influence of the functional groups is diluted as the role of CH_2_ groups becomes more relevant. The thermal transitions display an alternating or zigzag behavior, with PEAs that contain an even number of methylene groups in the diacid part displaying higher *T*_c_ and *T*_m_ values than the samples with an odd number of CH_2_ groups. This even–odd effect arises from the differences in the crystal packing of the chains depending on the even or odd number of CH_2_ groups in the diacid part, which is discussed in more detail in Section 3.3.^11,14,37–39^

**Figure 2:**
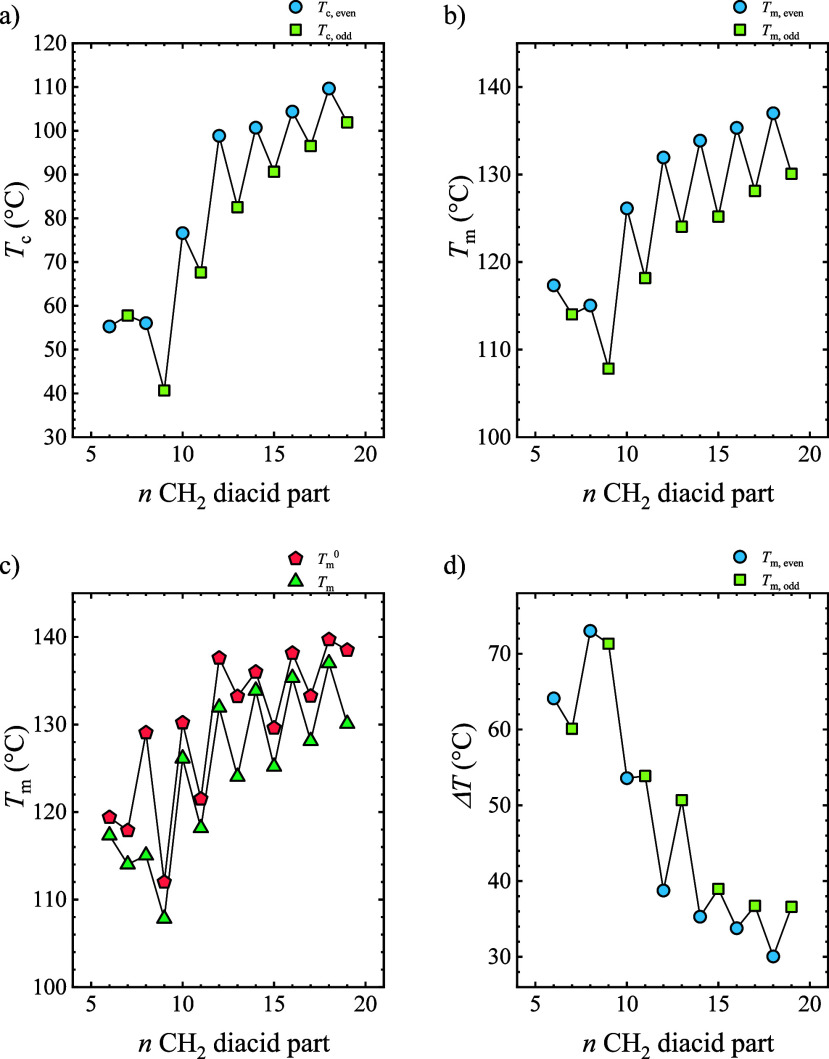
(a) Crystallization and (b) melting temperature of PEAs as a function of the number of methylene groups in the diacid part. (c) Comparison of the experimental melting temperature and the equilibrium melting temperature obtained from Hoffman–Weeks extrapolation. (d) Supercooling as a function of the number of methylene groups.

In this work, PEAs of up to 19 CH_2_ groups in the diacid part have been investigated for the first time, which is the largest number of methylene groups within the repeating unit reported. In this case, there is no saturation of the alternating behavior even if many methylene groups are introduced in the repeating unit. The results demonstrate that even when the poly­(ester amides) employed here have long alkyl chain lengths in their main backbone, the number of CH_2_ groups still plays a significant role in the crystalline structure and packing of the chains in the crystal, probably due to the strong hydrogen bonds between amide groups and carbonyl groups.

Interestingly, *T*_m_ only increases by around 30 °C across the series, which aligns with similar systems like polyesters^17^ or polyamides^25^ for a comparable increase of CH_2_ groups in the repeating unit. However, the impact of methylene groups is more dramatic at the crystallization temperatures. The supercooling (i.e., Δ*T* = *T*_m_^0^ *– T*_c_) has been estimated to analyze this effect more carefully. The *T*_m_^0^ value was obtained from the Hoffman–Weeks extrapolation procedure.^40^ As an example, the Hoffman–Weeks extrapolations of PEA8-8, PEA8-10, and PEA8-16 are included in the Figure S3. *T*_m_^0^ shows an alternating behavior similar to that seen in the *T*_m_ trend, with values generally higher than the experimental *T*_m_ values, as expected.

If the supercooling is calculated as Δ*T* = *T*_m_^0^ – *T*_c_, then an alternating behavior can be observed, except for the lowest number of methylene groups. The results indicate that PEAs with a low number of methylene groups require a higher supercooling to crystallize. By introducing methylene groups in the repeating unit, the supercooling drops from 75 to 30 °C. These results suggest that the presence of methylene groups enhances the flexibility of polymer chains, thereby facilitating the crystallization process. The increase of the flexibility with the number of CH_2_ groups is in line with the results reported in the literature; for example, for aliphatic polycarbonates, a reduction of *T*_g_ is observed with the increasing incorporation of CH_2_ groups in the repeating unit.^41,42^

The molar mass differences between the two polymer groups (medium molar mass, up to 13 CH_2_ groups, and low molar mass, above 14 CH_2_ groups) do not seem to affect the thermal properties since a smooth increase is observed with the number of CH_2_ in the repeating units. When the molar mass of a polymer is very small, a depression in the values of *T*_m_ and *T*_c_ higher than 30 °C with respect to higher molar mass materials is expected. Based on the trends obtained in Figure 2, we are confident that the samples are not in the low molar mass sensitive region and the chain end groups are not playing a relevant role in the thermal properties. Thus, it is fair to compare the thermal properties of the different samples without considering that the different molas masses may be affecting the even–odd trends.

The alternating trend was also observed in the crystallization and melting enthalpies (Figure 3). In this case, there are some deviations compared with the thermal transitions. The crystallinity percentage can be estimated by the equilibrium melting enthalpy. This Δ*H*_m_^0^ was estimated by the group contribution theory, which is a semiempirical approach developed by Van Krevelen; the values for each material are included in Table S1.^43^ If the crystalline percentage obtained from the melting scans is considered, the results show that PEAs have a crystallinity degree between 20 and 40% and that, in general, the increment of CH_2_ groups in the repeating unit facilitates the ability to crystallize. *X*_c_ can vary almost 10% just by introducing one methylene group, which reflects the relevance of studying the thermal properties since *X*_c_ impacts the final performance of polymers, including mechanical properties or permeability.^5–8^ The exceptions to the expected trend can arise from the possible differences in the crystalline structure and chain conformation.

**Figure 3:**
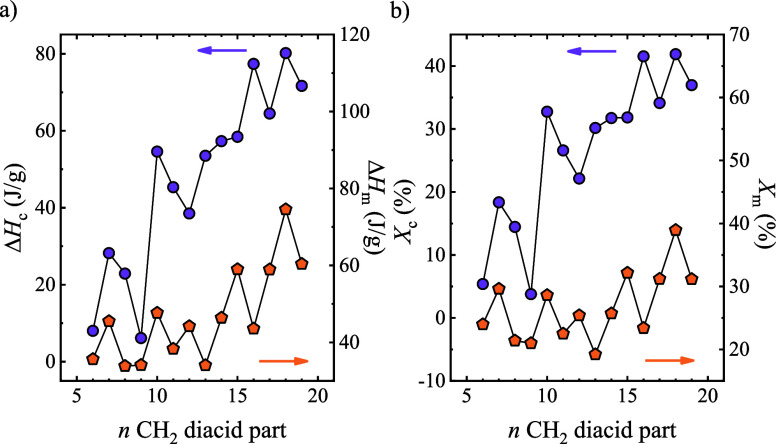
(a) Enthalpies obtained during cooling and subsequent heating scans as a function of the number of CH_2_ groups in the diacid part. (b) Crystallinity percentage of the materials, considering the crystallization and melting enthalpy.

### 3.2 Isothermal Crystallization Kinetics

Isothermal crystallization was studied by DSC to determine the overall crystallization kinetics, which comprise both nucleation and growth. The inverse of the half crystallization time (1/τ_50%_), proportional to the overall crystallization rate, increases as the crystallization temperature is reduced (Figure 4a). This indicates that crystallization is governed by nucleation (both primary and secondary nucleation, as the results are obtained by DSC) since the results correspond to the right-hand side of the usual bell-shaped curve, generally displayed when the overall crystallization rate is plotted as a function of *T*_c_.^44^ The crystallization rate was also plotted as a function of the supercooling (Figure 4b).

**Figure 4:**
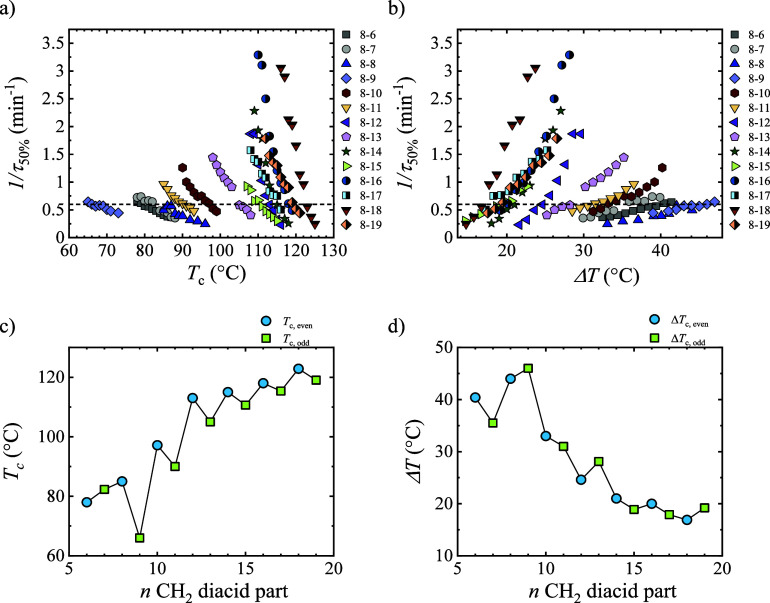
1/τ_50%_ as a function of (a) crystallization temperature and (b) supercooling for all PEAs. (c) Crystallization temperature and (d) supercooling required to reach 1/τ_50%_ = 0.6 min^–1^ value.

To compare the samples studied and ascertain the impact of the number of methylene groups on the crystallization kinetics, a constant crystallization rate has been considered, 0.6 min^–1^ (all the samples show this rate at different *T*_c_ values as can be seen in Figure 4a, indicated with the dotted line), and the *T*_c_ and Δ*T* necessary to reach that rate are compared (Figure 4c,d). Increasing the number of methylene groups, higher *T*_c_s, or lower supercoolings are needed to reach the selected rate. This means that the incorporation of methylene groups facilitates the crystallization process in accordance with nonisothermal experiments. In addition, an alternating behavior of *T*_c_ and Δ*T* is observed with the number of CH_2_ groups, requiring lower supercooling for the polymers with even numbers of CH_2_ groups. The alternating trend has been reported for a limited number of polymers, including polycarbonates^13^ and polyethers,^14^ which show a saturation regime with increasing CH_2_ number. These results have been related to the different crystalline packings of polymer chains depending on the even or odd number of CH_2_ groups. Since the number of CH_2_ affects the conformation that the chains adopt and the crystalline structure, it impacts the crystallization rate.^13–16^

The results indicate that the chain ends of our low molar mass samples do not seem to significantly affect the *T*_c_ value needed to reach a specific crystallization rate. When the molar mass is very low, the contribution of chain ends is important, and a drastic reduction in such a *T*_c_ value would be observed, which is not the case. Studies with polyesters such as poly­(caprolactone) have shown that above 2 kg/mol, the crystallization rate is practically constant.^45^

The data obtained by isothermal crystallization was analyzed employing the Avrami theory,^46–48^ thus obtaining the Avrami index, the overall crystallization rate constant, and the induction time for crystallization. For the PEAs investigated in this work, the Avrami index (*n*) values are between 2.5 and 4 (Figure 5a). The low *n* values in the range of 2.5–3.4 indicate that, as the index is close to 3,^33^ spherulites are nucleated instantaneously during crystallization. Avrami index values between 3.5 and 4 correspond to spherulites that nucleate sporadically,^33^ which usually results in polymer morphologies with spherulites of different sizes. For PEAs with CH_2_ numbers in the range of 6–11, there is no effect of even–odd CH_2_ groups. However, for PEAs with CH_2_ numbers above 12, the odd samples have lower Avrami indexes than even samples, with the even sample values close to 3.5 and the odd samples close to 3. This indicates that even samples tend to nucleate sporadically, whereas odd ones have more instantaneous nucleation. This result is relevant considering its impact on the morphology and that previous studies with polycarbonates and polyethers found no effect on the Avrami index of the number of CH_2_.

**Figure 5:**
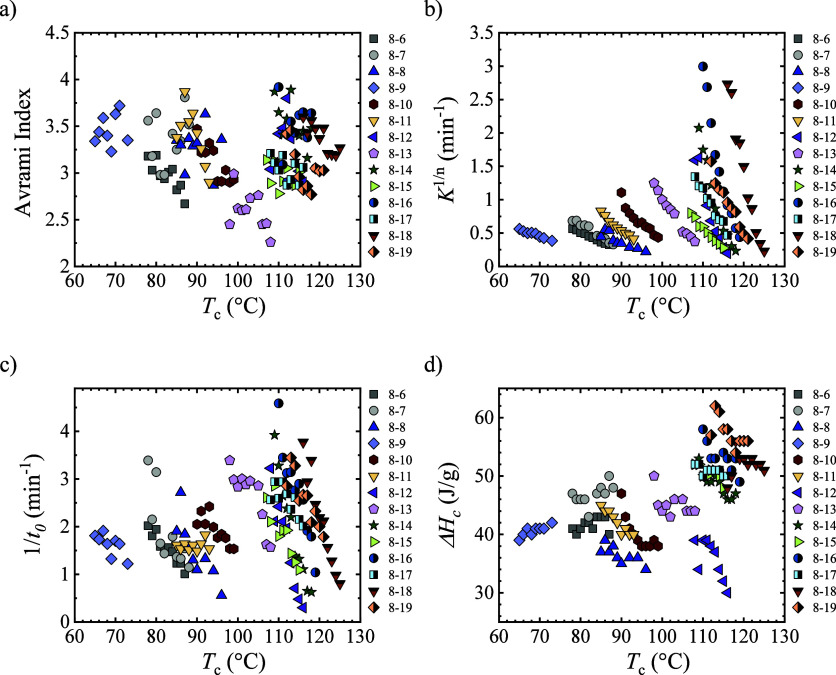
(a) Avrami index, (b) overall crystallization rate constant, (c) inverse of the induction time, and (d) crystallization enthalpy as a function of crystallization temperature.

Applying Avrami theory, the overall crystallization rate constant can be obtained, which depends on the Avrami index, as observed in the parameter units, i.e., min^–*n*^. Therefore, to compare the series studied in this work, we can elevate *K* to 1/*n*. The *K* ^1/*n*^ parameter shows a similar trend with *T*_c_ and Δ*T* compared to 1/τ_50%_ (Figure 5b). To analyze whether an even–odd effect is shown, a constant *K* ^1/*n*^ value is considered, 0.5 min^–1^, and the Δ*T* and *T*_c_ required to reach that value are compared (Figure S3a,b). The *T*_c_ increases with the CH_2_ number, showing an alternating trend similar to the trend in 1/τ_50%_. If Δ*T* is considered, the alternating trend is not so clear, but a decrease in supercooling with CH_2_ number is observed, reflecting that the introduction of CH_2_ facilitates crystallization.

The induction time, *t*_0_, obtained with the Avrami theory reflects the time needed for primary nucleation before any crystallization has started.^33,49,50^ The inverse of *t*_0_ is equivalent to the initial primary nucleation rate (Figure 5c). When considering the Δ*T* and *T*_c_ needed to reach a nucleation rate (or 1/*t*_0_) equal to 1.94 min^–1^, an alternating trend is observed (Figure S3c,d). Like other parameters derived from the Avrami theory, the supercooling required to reach that nucleation rate is reduced with the CH_2_ number. This implies that nucleation is facilitated with longer alkyl chains in the main backbone.

The crystallization enthalpy measured during the isothermal crystallization (Figure 5d) shows that the values are lower for even samples compared with the odd samples, except for 16 and 17 CH_2_ PEAs. It seems that for odd samples, it is easier to reach higher degrees of crystallinity. The trend is not in line with nonisothermal experiments since a different behavior was observed, but this could arise from the different conditions employed. During an isothermal experiment, the material crystallizes at a certain undercooling. Nucleation starts and crystal growth follows, and this allows the material to crystallize during the time it needs to complete its transformation to the semicrystalline state. Since high temperatures are used (well above the *T*_g_), the chains have enough mobility to develop the maximum crystallinity allowed (until it saturates) as a function of supercooling and crystallization time. However, during the nonisothermal process, the material experiences a continued increase in the undercooling, where the time spent at each crystallization temperature is dictated by the applied cooling rate. Therefore, once the material starts to crystallize, this crystallization process stops when the temperature is low enough to slow chain diffusion, so it is possible that the crystallization process does not reach the maximum saturation value for the studied polymer. A similar alternating trend in the crystallization enthalpies obtained during isothermal experiments has been reported for polyethers,^14^ with lower values for even samples than odd ones, although in that case, an odd–even effect was observed in the transition temperatures.

### 3.3 Structure and Morphology

#### 3.3.1 Microstructural Morphology Studied by PLOM

Polarized light optical microscopy was employed to correlate the even–odd effect with the semicrystalline morphology, as such a correlation has not been studied before for similar systems, as far as the authors are aware. The materials were heated to the appropriate temperature to remove the thermal history and were cooled at 20 °C/min to room temperature to allow the samples to crystallize. The micrographs obtained are shown in Figure 6. PEA8-6 and PEA8-9 show cold crystallization, so those materials were additionally heated to 90 and 80 °C to allow them to crystallize. The analysis of the images reveals that for PEA with a low number of methylene groups in the repeating unit, the nucleation density is very high, which results in significantly refined morphology. Spherulites can be clearly distinguished for PEAs with 13 or more methylene groups in the diacid part. The analysis of these seven samples (from PEA8-13 to PEA8-19) reveals that odd samples display sporadic nucleation since both large and small spherulites are observed in the micrographs. On the contrary, PEAs with an even number of CH_2_ (PEA8-14, PEA8-16, and PEA8-18) show a more homogeneous spherulite size, indicating instantaneous nucleation. A magnification of some of the materials (PEA8-14 to PEA8-19) is displayed in Figure S5. These results differ from the trend observed in isothermal crystallization, as nucleation in nonisothermal conditions occurs continuously during cooling in a wide range of temperatures; thus, the changes can be attributed to the very different thermal history of the samples.

**Figure 6:**
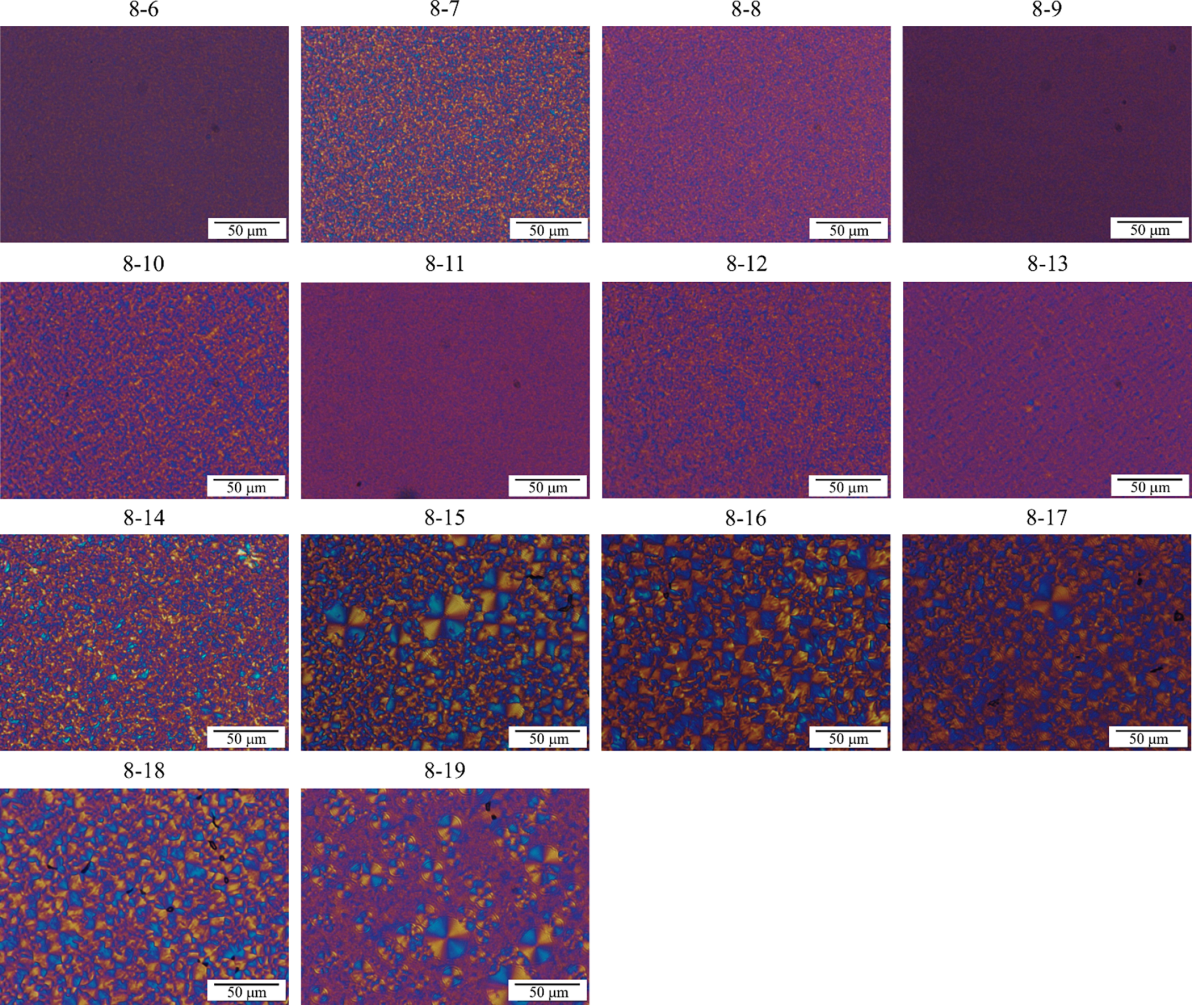
PLOM micrographs acquired after cooling the samples nonisothermally from a homogeneous melt state at 20 °C/min.

The spherulites of the analyzed PEAs are negative, as the first and third quadrants are yellow when using a red-sensitive plate,^51^ which is typical for most polymers. Usually, the refractive index along the chain axis is larger than that in the direction perpendicular to the chain. As crystalline lamellae grow radially and chains are folded within the lamellae, the chains will be placed tangentially to the radius of the spherulite.^51,52^ Having an odd or even number of methylene groups in the repeating unit of the chains does not modify the refractive index trend, the value of which is highest along the chain.

A closer look at the spherulites reveals that even PEAs have the typical Maltese Cross extinction pattern, whereas the odd samples exhibit banding. The source of banding in spherulites is discussed in the literature. It has been mainly related to lamellar twisting but also to periodic height changes or periodic modification of the lamellae orientation and size, among other possibilities.^51–54^ Our results indicate that having an odd number of CH_2_ groups promotes the appearance of banded spherulites, affecting some of the mentioned parameters, which is not observed with even samples.

Overall, the study of the morphology at the microscopic level reveals for the first time the implications of even and odd numbers of methylene groups in the repeating units of PEAs. Having even or odd numbers of CH_2_ groups impacts the nucleation mechanism (i.e., instantaneous vs sporadic) and the microstructure of the spherulites (i.e., the appearance of ring-banded spherulites in the odd samples). However, it does not affect the refractive index along the chain, as all spherulites obtained are negative, regardless of the even–odd effect.

#### 3.3.2 Long Period and Crystalline Structure Studied by SAXS and WAXS

##### Long Period

SAXS experiments were carried out at the synchrotron; see the experimental part. Figure 7 shows the Lorentz corrected SAXS patterns, i.e., *Iq* ^2^ vs *q*. The peak appearing at the lowest *q* values is due to the long period, d*, which comprises the crystalline lamellae and amorphous layer thickness. The following section depicts and discusses the peaks at higher *q* values. The SAXS patterns show a shift of the maximum to lower *q* values, from 0.77 to 0.49 nm^–1^, which indicates an increase in the long period with the number of methylene groups in the diacid part of PEA.

**Figure 7:**
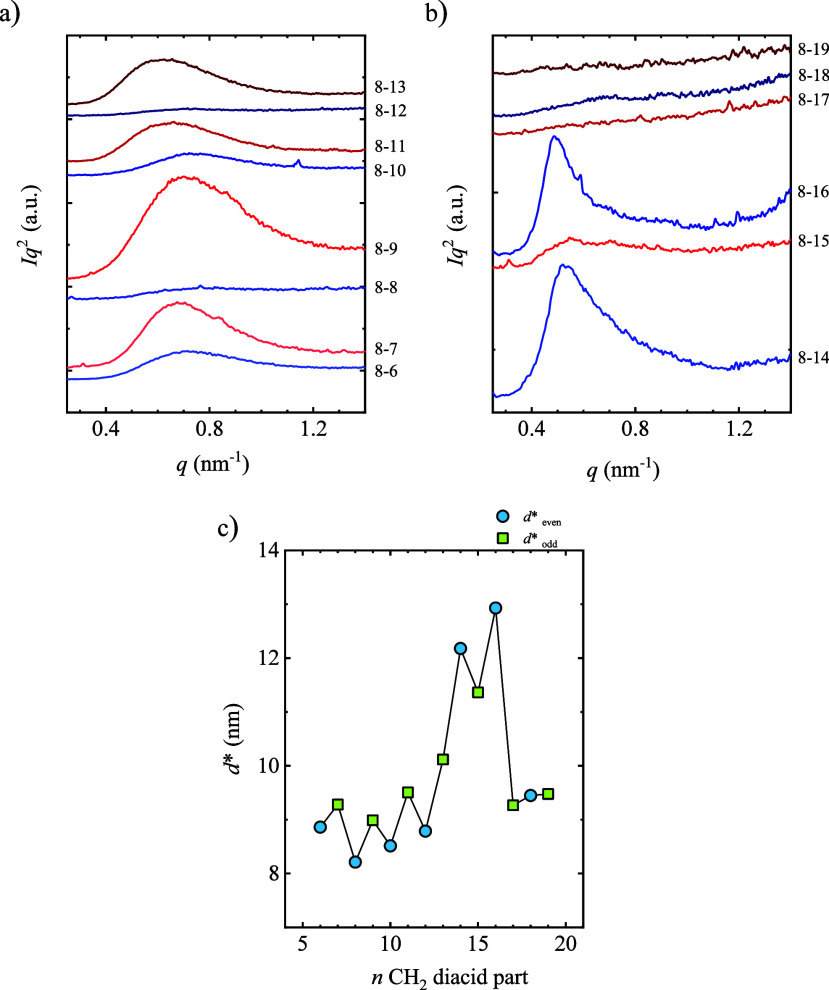
Lorentz-corrected SAXS patterns at room temperature for PEAs with (a) 6–13 CH_2_ groups in the diacid part and (b) 14–19 CH_2_ groups after obtaining a standard crystalline state by cooling at 20 °C/min from the melt for PEA samples. (c) Long period values, d*, as a function of the number of methylene groups in the diacid part.

Although the intensity of the peaks is low for some PEAs, from Bragg′s law, d* = 2 π/*q*, the long period can be estimated.^55^ Figure 7c shows the obtained long period as a function of the number of CH_2_ groups in the diacid part. The long period values increase with the number of methylene groups from 8 to 13 nm, while they increase drastically for a few samples (i.e., for *n*CH_2_ equal to 14, 15, and 16). This result cannot be due to the low molar mass samples of PEA8–14 and beyond since a long period reduction has generally been observed in polyesters as the molar mass decreases.^43^ Interestingly, the odd samples show longer period values than the even ones with some exceptions at high *n*CH_2_. However, the contrary trend is observed in the thermal properties (i.e., higher *T*_m_ for even samples than for odd samples). Considering the samples’ long period and crystallinity degree (see Section 2 for details), it is possible to estimate the crystalline lamellar thickness and amorphous layer thickness. Although the *X*_c_ values obtained from this method are small (indicating that they are probably underestimated), it is possible to compare them qualitatively. The crystalline and amorphous layers display a fluctuation in the values with the even–odd number of methylene groups; see Figure S6 in the Supporting Information. The amorphous layer is larger than the crystalline layer. Thus, the results indicate that having an even or odd number of CH_2_ groups impacts the structure of semicrystalline materials at the nanometric level.

##### Crystalline Structure

The crystalline structure of the materials was investigated by WAXS. For that, WAXS patterns were acquired at room temperature after applying the thermal procedure explained in the experimental section. The poly­(ester amide)­s show a complex pattern (Figure 8) with two to three main reflections and several overlapping peaks in the *q* region of 10–20 nm^–1^. Strong reflections are displayed in the low *q* region (<10 nm^–1^). If the medium *q* region is analyzed (10–20 nm^–1^), it can be observed that the most intense reflection is located at around 15 nm^–1^. In literature, Puiggalí et al. have investigated the crystalline structure of several poly­(ester amide)­s,^56,57^ including PEA8-4 and PEA8-10.^57^ PEA8-4 has a triclinic unit cell with cell dimensions of *a* = 0.475 nm, *b* = 1.35 nm, and *c* = 2.26 nm and the angles α = 90°, β = 77°, and γ = 64°. PEA8-6 and PEA 8-8 have a main reflection at around 15.2 nm^–1^ (Figure 8), which can be assigned to the (100) reflection of the PEA8-4-type unit cell, assuming that those samples adopt a triclinic unit cell. Two weak reflections at 13.5 and 17 nm^–1^ can be correlated with the (110) and (130) planes. However, for PEA8-10, our X-ray diffraction data are very different from that of Puiggalí et al.,^57^ which reported a monoclinic unit cell with dimensions as follows: *a* = 0.479 nm, *b* = 1.832 nm, and *c* = 2.88 nm, β = 77°. This is presumably due to the existence of polymorphism in PEA8-10. No crystallographic data are available for the rest of the samples. Therefore, it is not possible to index them. Despite that, the WAXS curves in the 10–20 nm^–1^ range correspond to the characteristic spacing of interchain crystal packing, i.e., (*hk*0) reflections. Considering the similarity of the samples, it is reasonable to assume that the strongest reflection peak belongs to the same lattice plane.

**Figure 8:**
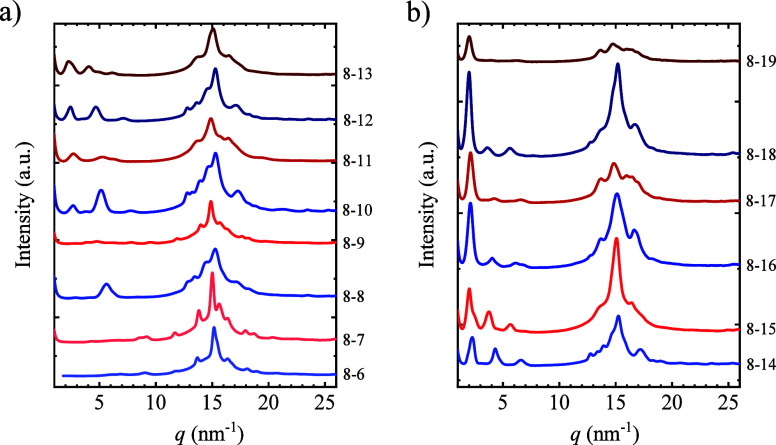
WAXS patterns acquired at room temperature for the poly­(ester amide)­s investigated in this work after applying an appropriate thermal procedure: (a) PEA with 6 to 13 CH_2_ groups in the diacid part and (b) 14 to 19 CH_2_ groups.

The analysis of the low *q* region peaks (2–10 nm^–1^) accessible with WAXS shows an even–odd effect with the number of methylene groups; see Figure 9a,b. Those peaks have been indexed as 001, 002, and 003 from low to high *q* values. The *d*-spacing has been estimated using Bragg′s law. All the peaks show a reduction in *q* value and correspondingly an increase of *d*-spacing with the number of methylene groups, as could be expected with the increase in the repeating unit’s size. Fluctuation in the spacing of (00*l*) is also observed, although the variation is less regular. This indicates that the α and β lattice angles must have an even–odd effect because the *c*-axis is expected to increase monotonically with the number of methylene groups.

**Figure 9:**
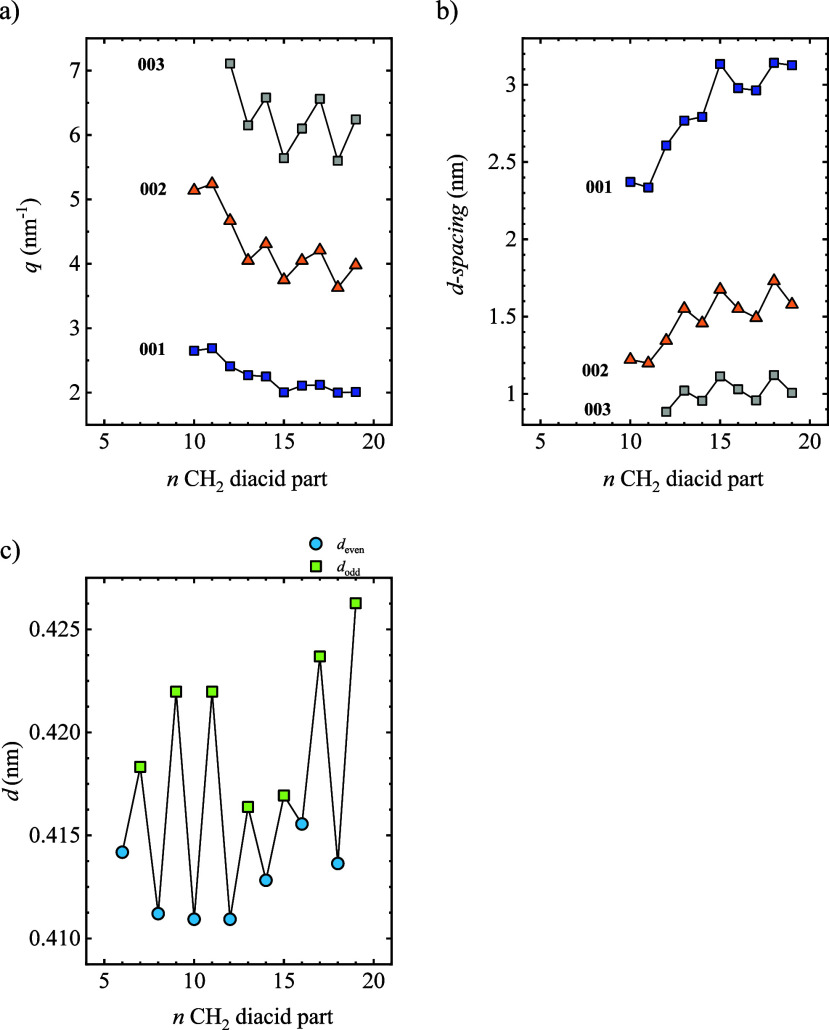
(a) *q* values and (b) *d*-spacings of the three peaks appearing at a low *q* value region in the WAXS as a function of the number of methylene groups in the diacid part. (c) *d*-spacings calculated from the main reflection as a function of the number of CH_2_ groups in the diacid part. Note that the indexes of the peaks in different samples may be different.

The *d*-spacing of the strongest peak in the *q* range of 10–20 nm^–1^ is shown in Figure 9c (the *q* value is shown in Figure S7). PEAs with an odd number of CH_2_ groups have a higher *d*-spacing value than even samples, which could indicate a denser crystal packing of the chains for the even samples. There is no monotonic trend of *d*-spacing with the number of CH_2_ groups. This is because the strongest reflection in the different samples may correspond to different planes. Samples with 8–12 methylene groups have almost a constant *d* value, with higher values for odd samples. For PEAs with 13 CH_2_ groups, the *d* spacing increases with the number of methylene groups.

The differences in the structure for even and odd samples arise from the samples’ different chain conformations and intermolecular interactions. Since PEAs have amide groups that can form strong hydrogen bonds, those are the most relevant interactions that will determine the crystal packing of the chains. Indeed, it is well known that hydrogen bonds have a decisive role in the crystal packing of the chains of polyamides.^25^ The ability to form hydrogen bonds changes significantly depending on whether the material has an even or odd number of methylene groups. Puiggalí et al. have suggested that polymer chains of PEA8-10 are shifted along the main chain direction so that NH groups face carbonyl groups of the adjacent chains.^57^ In this way, hydrogen bond formation is favored, resulting in better crystal packing of the chains. On the contrary, PEAs with an odd number of CH_2_ in the repeating unit cannot form as many hydrogen bonds as even samples. In addition, there could be some steric hindrance from the carbonyl groups.^32^ Thus, crystal packing of the chains is less effective for odd samples, resulting in lower thermal properties. This demonstrates that the even–odd effect in PEAs originates from the different crystal packing of the chains, which has not been observed before for this polymer family.

## 4 Conclusions

The relationship between thermal properties and poly­(ester amide) structures with varying methylene groups in the diacid part was investigated. The study covers, for the first time, PEAs containing long alkyl chain lengths in the repeating unit, demonstrating that an even–odd effect persists even with 19 CH_2_ groups in the diacid part. This means that in poly­(ester amide)­s, the properties of the material fluctuate significantly even when they include long alkyl chains, thus allowing for tunable material properties (e.g., crystallization, melting points, crystallinity, and morphology). This is the system with the largest alkyl chain lengths in the repeating unit reported to show this effect, which is relevant considering its impact on the final performance of the material. The melting and crystallization temperatures obtained under nonisothermal conditions are higher for PEAs with an even number of CH_2_ groups than for odd PEAs. A similar alternating trend is obtained during the isothermal crystallization. We demonstrate that the even–odd effect arises from the differences in the crystal packing of the chains, which has not been proved before for poly­(ester amide)­s. The packing of the PEAs may be governed by the strong hydrogen bonds provided by amide groups, which can induce differences in the structure, even when they have very large alkyl chain lengths between the functional groups. For the first time, the impact of an even or odd number of CH_2_ on the microstructure and spherulitic morphology is reported, which determines the size of spherulites and the appearance of banding. Overall, this work shows that by carefully designing polymer systems with tailored intermolecular interactions considering the functional groups and the alkyl chain length, it is possible to fine-tune the thermal properties of polymers, which opens the door to controlling the final performance of semicrystalline materials.

## Supplementary Material



## Notes

The molar mass measurement conditions, SEC curves, and a comment on the molar mass of the samples; WAXS curves obtained as a function of temperature for several PEAs; melting temperature as a function of crystallization temperature displayed for several PEAs to show how *T*_m_^0^ is obtained; data from Van Krevelen group contribution theory to obtain the equilibrium melting enthalpy; crystallization temperature and supercooling needed to reach a constant overall crystallization rate and induction time; the estimated crystalline lamellar thickness and amorphous layer thickness; and *q* values of the main reflection as a function of CH_2_ groups (PDF)

## References

[1] Zhu Y., Romain C., Williams C. K. (2016). Sustainable polymers from renewable
resources. Nature.

[2] Schneiderman D. K., Hillmyer M. A. (2017). 50th anniversary
perspective: there is a great future
in sustainable polymers. Macromolecules.

[3] Rodriguez-Galan A., Franco L., Puiggali J. (2011). Degradable
poly (ester amide) s for
biomedical applications. Polymers.

[4] Lovinger, A. J. Foreword in Phase Transitions in Polymers: The Rule of Metastable States; Elsevier: Amsterdam, The Netherlands, 2008 DOI: 10.1016/B978-0-444-51911-5.X0001-1.

[5] Pantani R., Sorrentino A. (2013). Influence
of crystallinity on the biodegradation rate
of injection-moulded poly (lactic acid) samples in controlled composting
conditions. Polym. Degrad. Stab..

[6] Kundu P. P., Biswas J., Kim H., Choe S. (2003). Influence of film preparation
procedures on the crystallinity, morphology and mechanical properties
of LLDPE films. Eur. Polym. J..

[7] Drieskens M., Peeters R., Mullens J., Franco D., Lemstra P. J., Hristova-Bogaerds D. G. (2009). Structure
versus properties relationship of poly (lactic
acid). I. Effect of crystallinity on barrier properties. Journal of Polymer Science Part B: Polymer Physics.

[8] Compañ V., Del Castillo L. F., Hernández S. I., López-González M. M., Riande E. (2010). Crystallinity effect on the gas transport in semicrystalline
coextruded films based on linear low density polyethylene. Journal of Polymer Science Part B: Polymer Physics.

[9] Piorkowska, E. ; Rutledge, G. C. Handbook of Polymer Crystallization; John Wiley & Sons, Inc.: Hoboken, New Jersey, US, 2013 DOI: 10.1002/9781118541838

[10] Auriemma, F. ; Alfonso, G. C. ; de Rosa, C. Polymer Crystallization I. From Chain Microstructure to Processing; Springer Cham: Cham, Switzerland, 2017 DOI: 10.1007/978-3-319-49203-2

[11] Stempfle F., Ortmann P., Mecking S. (2016). Long-chain
aliphatic polymers to
bridge the gap between semicrystalline polyolefins and traditional
polycondensates. Chemical reviews.

[12] Rizzarelli P., Cirica M., Pastorelli G., Puglisi C., Valenti G. (2015). Aliphatic
poly (ester amide) s from sebacic acid and aminoalcohols of different
chain length: Synthesis, characterization and soil burial degradation. Polym. Degrad. Stab..

[13] Pérez-Camargo R. A., Meabe L., Liu G., Sardon H., Zhao Y., Wang D., Müller A. J. (2021). Even–odd
effect in aliphatic
polycarbonates with different chain lengths: From poly (hexamethylene
carbonate) to poly (dodecamethylene carbonate). Macromolecules.

[14] Flores I., Pérez-Camargo R. A., Gabirondo E., Caputo M. R., Liu G., Wang D., Sardon H., Muller A. J. (2022). Unexpected structural properties in the saturation
region of the odd–even effects in aliphatic polyethers: Influence
of crystallization conditions. Macromolecules.

[15] Soccio M., Lotti N., Finelli L., Gazzano M., Munari A. (2007). Aliphatic
poly (propylene dicarboxylate) s: Effect of chain length on thermal
properties and crystallization kinetics. Polymer.

[16] Papageorgiou G. Z., Bikiaris D. N. (2005). Crystallization
and melting behavior of three biodegradable
poly (alkylene succinates). A comparative study.
Polymer.

[17] Zhou C., Wei Z., Yu Y., Shao S., Leng X., Wang Y., Li Y. (2019). Biobased long-chain aliphatic polyesters of 1, 12-dodecanedioic acid
with a variety of diols: Odd-even effect and mechanical properties. Materials Today Communications.

[18] Prisacariu C., Scortanu E. (2011). Influence of the type
of chain extender and urethane
group content on the mechanical properties of polyurethane elastomers
with flexible hard segments. High Performance
Polymers.

[19] Egbe D. A. M., Ulbricht C., Orgis T., Carbonnier B., Kietzke T., Peip M., Metzner M., Greicke M., Birckner E., Pakula T., Neher D., Grummt U.-W. (2005). Odd–Even
Effects and the Influence of Length and Specific Positioning of Alkoxy
Side Chains on the Optical Properties of PPE–PPV Polymers. Chem. Mater..

[20] Tasaki M., Yamamoto H., Yoshioka T., Hanesaka M., Ninh T. H., Tashiro K., Jeon H. J., Choi K. B., Jeong H. S., Song H. H., Ree M. H. (2014). Crystal
structure analyses of arylate
polyesters with long methylene segments and their model compounds
on the basis of 2-D X-ray diffractions and infrared progression bands. Polymer.

[21] Tasaki M., Yamamoto H., Yoshioka T., Hanesaka M., Ninh T. H., Tashiro K., Jeon H. J., Choi K. B., Jeong H. S., Song H. H., Ree M. H. (2014). Microscopically-viewed
relationship
between the chain conformation and ultimate Young’s modulus
of a series of arylate polyesters with long methylene segments. Polymer.

[22] Zhang X., Zuo X., Ortmann P., Mecking S., Alamo R. G. (2019). Crystallization
of Long-Spaced Precision Polyacetals I: Melting and Recrystallization
of Rapidly Formed Crystallites. Macromolecules.

[23] Coffman D. D., Berchet G. J., Peterson W. R., Spanagel E. W. (1947). Polymeric amides
from diamines and dibasic acids. J. Polym. Sci..

[24] Kinoshita Y. (1959). An investigation
of the structures of polyamide series. Die Makromolekulare
Chemie: Macromolecular Chemistry and Physics.

[25] Rulkens, R. ; Koning, C. E. Chemistry and technology of polyamides. In Polymer science: a comprehensive reference; Elsevier, 2012 DOI:10.1016/B978-0-444-53349-4.00147-3

[26] Fernández C. E., Bermudez M., Versteegen R. M., Meijer E. W., Muller A. J., Muñoz-Guerra S. (2009). Crystallization
studies on linear aliphatic n-polyurethanes. J. Polym. Sci., Part B: Polym. Phys..

[27] Jang Y.-J., Sangroniz L., Hillmyer M. A. (2022). Ductile gas barrier poly (ester-amide)­s
derived from glycolide. Polymer Chemistry.

[28] Sangroniz L., Jang Y.-J., Hillmyer M. A., Müller A. J. (2023). Tuning
the crystallization and thermal properties of polyesters by introducing
functional groups that induce intermolecular interactions. J. Chem. Phys..

[29] Sangroniz L., Jang Y.-J., Hillmyer M. A., Müller A. J. (2022). The role
of intermolecular interactions on melt memory and thermal fractionation
of semicrystalline polymers. The Journal of
Chemical Physics.

[30] Koyama E., Sanda F., Endo T. (1997). Polycondensations of dicarboxylic
acids and diols derived from optically active amino alcohols. Journal of Polymer Science Part A: Polymer Chemistry.

[31] Fey T., Keul H., Höcker H. (2003). Interconversion
of Alternating Poly
(ester amide) s and Cyclic Ester Amides from Adipic Anhydride and
α, ω-Amino Alcohols. Macromol. Chem.
Phys..

[32] Tetsuka H., Doi Y., Abe H. (2006). Synthesis and Thermal Properties of Novel Periodic
Poly (ester– amide) s Derived from Adipate, Butane-1, 4-diamine,
and Linear Aliphatic Diols. Macromolecules.

[33] Lorenzo A. T., Müller A. J. (2008). Estimation
of the Nucleation and Crystal Growth Contributions
to the Overall Crystallization Energy Barrier. J. Polym. Sci., Part B: Polym. Phys..

[34] Pérez-Camargo R. A., Liu G., Wang D., Müller A. J. (2022). Experimental and Data Fitting Guidelines
for the Determination of Polymer Crystallization Kinetics. Chin. J. Polym. Sci..

[35] Kluge M., Papadopolous L., Magaziotis A., Tzetzis D., Zamboulis A., Bikiaris D. N., Robert T. (2020). A Facile Method
to Synthesize Semicrystalline
Poly­(ester amide)­s from 2,5-Furandicarboxylic Acid, 1,10-Decanediol,
and Crystallizable Amido Diols. ACS Sustainable
Chem. Eng..

[36] Wunderlich B. (1995). Calorimetry
and thermal analysis of polymers. J. Therm.
Anal..

[37] Baeyer A. (1877). Ueber regelmässigkeiten
im schmelzpunkt homologer verbindungen. Berichte
der deutschen chemischen Gesellschaft.

[38] Boese R., Weiss H. C., Bläser D. (1999). The Melting
point alternation in
the short-chain n-alkanes: single-crystal x-ray analyses of propane
at 30 K and of n-butane to n-nonane at 90 K. Angewandte Chemie International Edition.

[39] Yang K., Cai Z., Jaiswal A., Tyagi M., Moore J. S., Zhang Y. (2016). Dynamic Odd–Even
Effect in Liquid n-Alkanes near Their Melting Points. Angewandte Chemie International Edition.

[40] Hoffman J. D., Weeks J. J. (1962). Melting process
and the equilibrium melting temperature
of polychlorotrifluoroethylene. J. Res. Natl.
Bur. Stand. Sect. A.

[41] Meabe L., Lago N., Rubatat L., Li C., Müller A. J., Sardon H., Armand M., Mecerreyes D. (2017). Polycondensation
as a versatile synthetic route to aliphatic polycarbonates for solid
polymer electrolytes. Electrochim. Acta.

[42] Chee K. K. (1991). Dependence
of glass transition temperature on chain flexibility and intermolecular
interactions in polymers. J. Appl. Polym. Sci..

[43] Van Krevelen, D. W. Properties of Polymers, 3rd ed.; Van Krevelen, D. W. , Ed.; Elsevier: Amsterdam, 1997. 10.1016/C2009-0-05459-2

[44] Schultz, J. M. Polymer crystallization: the development of crystalline order in thermoplastic polymers; American Chemical Society, Oxford University Press: New York, 2001.

[45] Fernández-Tena A., Pérez-Camargo R. A., Coulembier O., Sangroniz L., Aranburu N., Guerrica-Echevarria G., Liu G., Wang D., Cavallo D., Müller A. J. (2023). Effect
of Molecular Weight on the Crystallization and Melt Memory of Poly
(ε-caprolactone)­(PCL). Macromolecules.

[46] Reiter, G. ; Strobl, G. R. Progress in Understanding of Polymer Crystallization; Springer Berlin: Heidelberg, 2007 DOI:10.1007/3-540-47307-6

[47] Avrami M. (1941). Granulation,
Phase Change, and Microstructure Kinetics of Phase Change. III. J. Chem. Phys..

[48] Avrami M. (1940). Kinetics of
Phase Change. II Transformation-time Relations for Random Distribution
of Nuclei. J. Chem. Phys..

[49] Imai M., Mori K., Mizukami T., Kaji K., Kanaya T. (1992). Structural
formation of poly (ethylene terephthalate) during the induction period
of crystallization: 2. Kinetic analysis based on the theories of phase
separation. Polymer.

[50] Baltá
Calleja F. J., Ezquerra T. A. (2001). Polymer Crystallization: General Concepts
of Theory and Experiment.

[51] Wunderlich, B. Macromolecular physics; Academic Press: New York, NY, 1973, Vol. 1 DOI: 10.1016/B978-0-12-765601-4.X5001-X

[52] Crist B., Schultz J. M. (2016). Polymer spherulites:
A critical review. Prog. Polym. Sci..

[53] Woo E. M., Lugito G. (2015). Origins of periodic
bands in polymer spherulites. Eur. Polym. J..

[54] Lovinger A. J. (2020). Twisted
crystals and the origin of banding in spherulites of semicrystalline
polymers. Macromolecules.

[55] Elton L. R., Jackson D. F. (1966). X-ray diffraction
and the Bragg law. Am. J. Phys..

[56] Botines E., Teresa Casas M, Puiggalí J. (2007). Alternating poly­(ester amide)­s of
glycolic acid and ω-amino acids: Crystalline morphology and
main crystallographic data. J. Polym. Sci. B
Polym. Phys..

[57] Casas M. T., Puiggalí J. (2009). Crystalline
structure of sequential poly­(ester amide)­s
derived from glycolic acid, 1,6-hexanediamine, and even aliphatic
dicarboxylic acids. J. Polym. Sci. B Polym.
Phys..

