# Supplementary material for: Strong Hydrogen Bonds Sustain Even–Odd Effects in Poly(ester amide)s with Long Alkyl Chain Length in the Backbone
Source: Biomacromolecules. 2024 Oct 30;25(11):7500–10. doi: 10.1021/acs.biomac.4c01191 (PMC12884464; doi:10.1021/acs.biomac.4c01191)
Supplement: Supplementary file 1 [file bm4c01191_si_001.pdf]

Supporting Information

Strong hydrogen bonds sustain even-odd effects in poly(ester amide)s with long alkyl chains length in the backbone

Leire Sangroniz[1,2*], Jorge L. Olmedo-Martínez[2], Wenxian Hu[3,4], Yoon-Jung Jang[1], Guoming Liu[3,4], Marc A. Hillmyer[1], Alejandro J. Müller[2,5*]

[1]Department of Chemistry, University of Minnesota, Minneapolis, MN55455-0431, US.

[2]POLYMAT and Department of Polymers and Advanced Materials: Physics, Chemistry and Technology, Faculty of Chemistry, University of the Basque Country UPV/EHU, Paseo Manuel de Lardizábal, 3, 20018 Donostia-San Sebastián, Spain.

[3]Beijing National Laboratory for Molecular Sciences, CAS Key Laboratory of Engineering Plastics, Institute of Chemistry, Chinese Academy of Sciences, Beijing 100190, China.

[4]University of Chinese Academy of Sciences, Beijing 100049, China;

[5]IKERBASQUE, Basque Foundation for Science, Plaza Euskadi 5, 48009, Bilbao, Spain.

Molar mass measurements

The molar mass of PEAs was measured by size exclusion chromatography in an EcoSEC SEC system HCL-8240GPC Tosoh. A refractive index detector was used. 1,1,1,3,3,3-hexafluorosiopropanol (HFIP) with a 0.025 M concentration of potassium trifluoroacetate was employed as the mobile phase. 0.3 mL min^{-1} flow rate was used and the experiments were performed at 40 °C. The SEC instrument was calibrated previously employing poly(methyl methacrylate) standards from PolyAnalytik.

Molar mass comment

The number average molar mass as a function of the number of methylene groups in the diacid part is shown in Figure S1a. The samples can be divided in two groups according to the molar mass, as has been mentioned in the main manuscript. Considering the large dispersity of low molar mass samples (PEA8-14 to PEA8-19) the SEC curves are displayed in Figure S1b and S1c to compare the results. From these plots it can be seen that the broadness of the peak does not change significantly among the materials.

In addition, the thermal properties can be considered, which seem to indicate that the samples are above or just around the entanglement molar mass since there is no a reduction of thermal properties for the low molar mass samples. It should be considered that for other polymers such as poly(ε-caprolactone) samples with molar masses below 2 kg/mol exhibit a depression in the melting and crystallization temperature close to 30 °C.[1] In this work a smooth trend is observed in thermal properties with n CH_2 which does not reflect the differences in molar mass between the two sets of samples. If the molar mass had a significant impact, an important drop in the T_m should have been observed for the lower molar mass samples. However, we observe an increase in T_m without any disruptions in the 13-14 CH_2 region. Thus, we consider that is fair to compare the results of this series of PEAs.

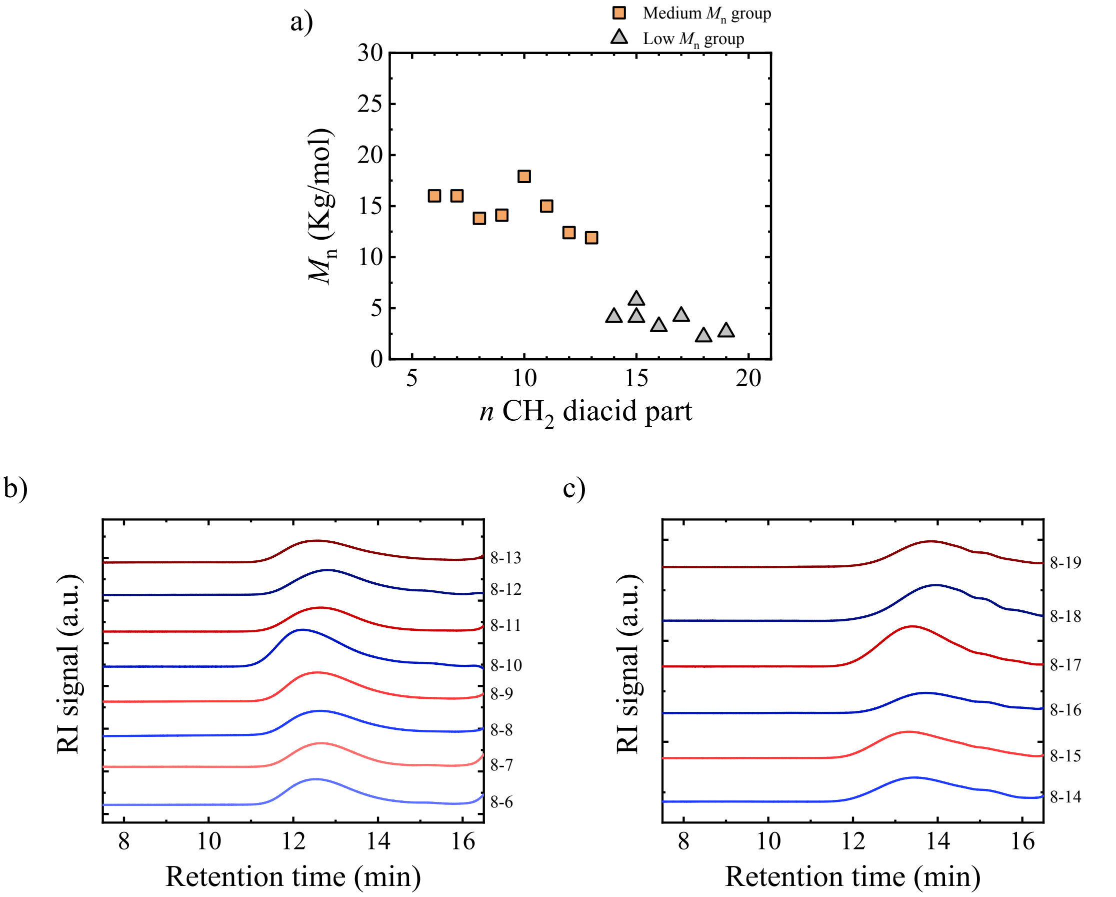

Figure S1. a) Number average molar mass as a function of the number of methylene groups in the diacid part. SEC curves for b) medium molar mass group (PEA8-6 to PEA8-13) and c) low molar mass group (PEA8-14 to PEA8-19).

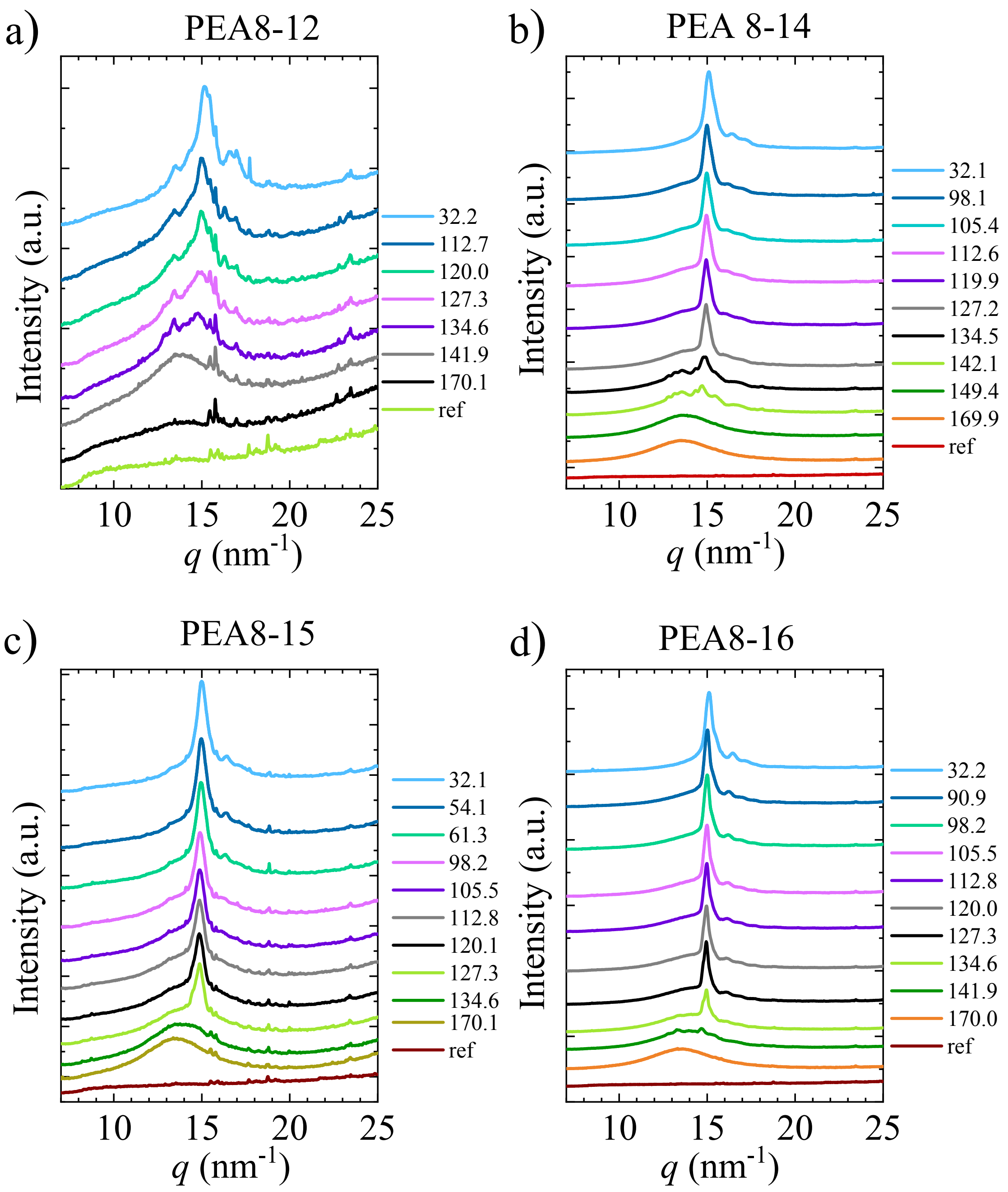
a)
PEA8-12
Intensity (a.u.)
q (nm-1)
10
15
20
25
32.2
112.7
120.0
127.3
134.6
141.9
170.1
ref
b)
PEA 8-14
Intensity (a.u.)
q (nm-1)
10
15
20
25
32.1
98.1
105.4
112.6
119.9
127.2
134.5
142.1
149.4
169.9
ref
c)
PEA8-15
Intensity (a.u.)
q (nm-1)
10
15
20
25
32.1
54.1
61.3
98.2
105.5
112.8
120.1
127.3
134.6
170.1
ref
d)
PEA8-16
Intensity (a.u.)
q (nm-1)
10
15
20
25
32.2
90.9
98.2
105.5
112.8
120.0
127.3
134.6
141.9
170.0
ref

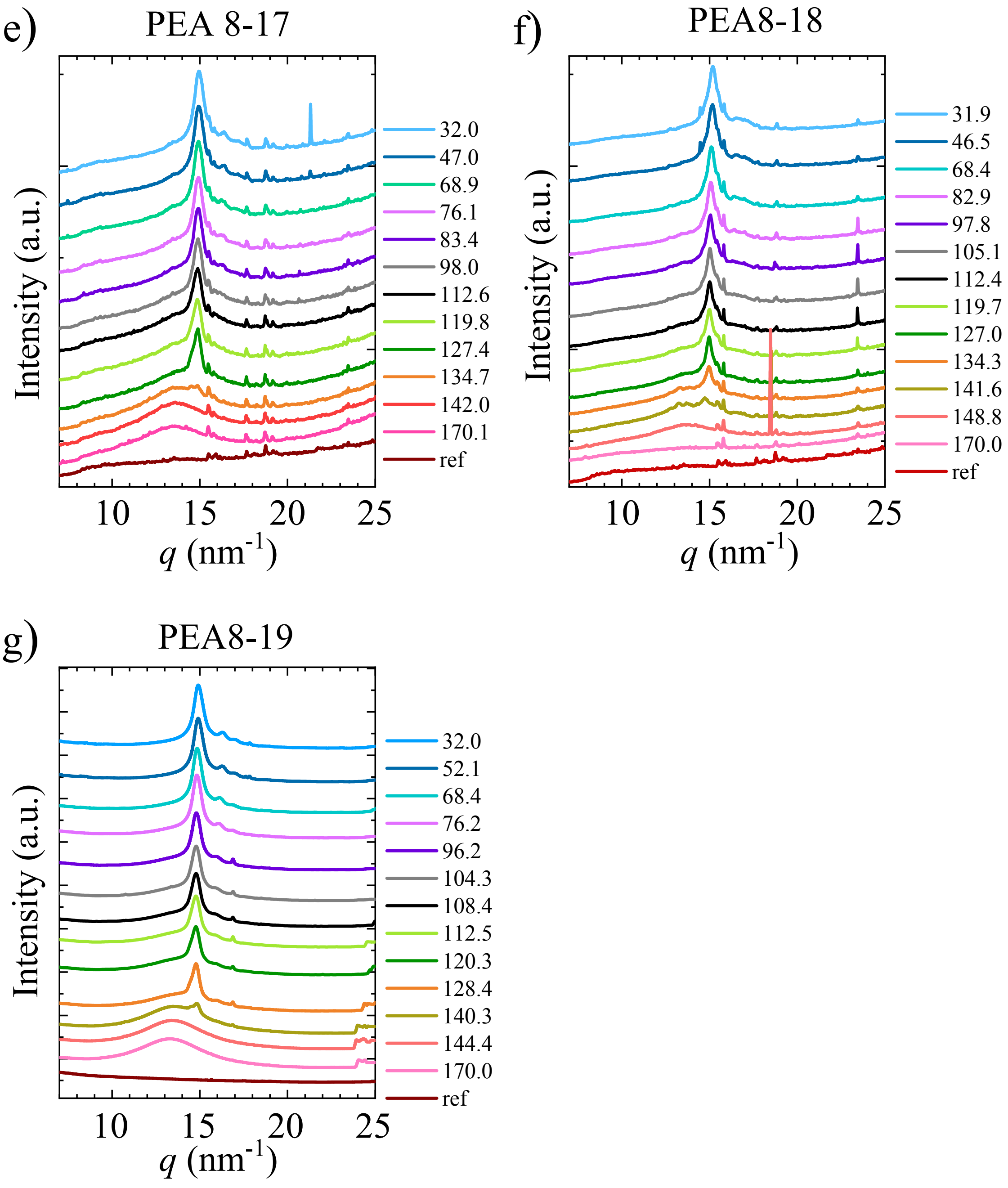

Figure S2. WAXS patterns acquired during heating at 20 °C/min after crystallizing the samples in the DSC.

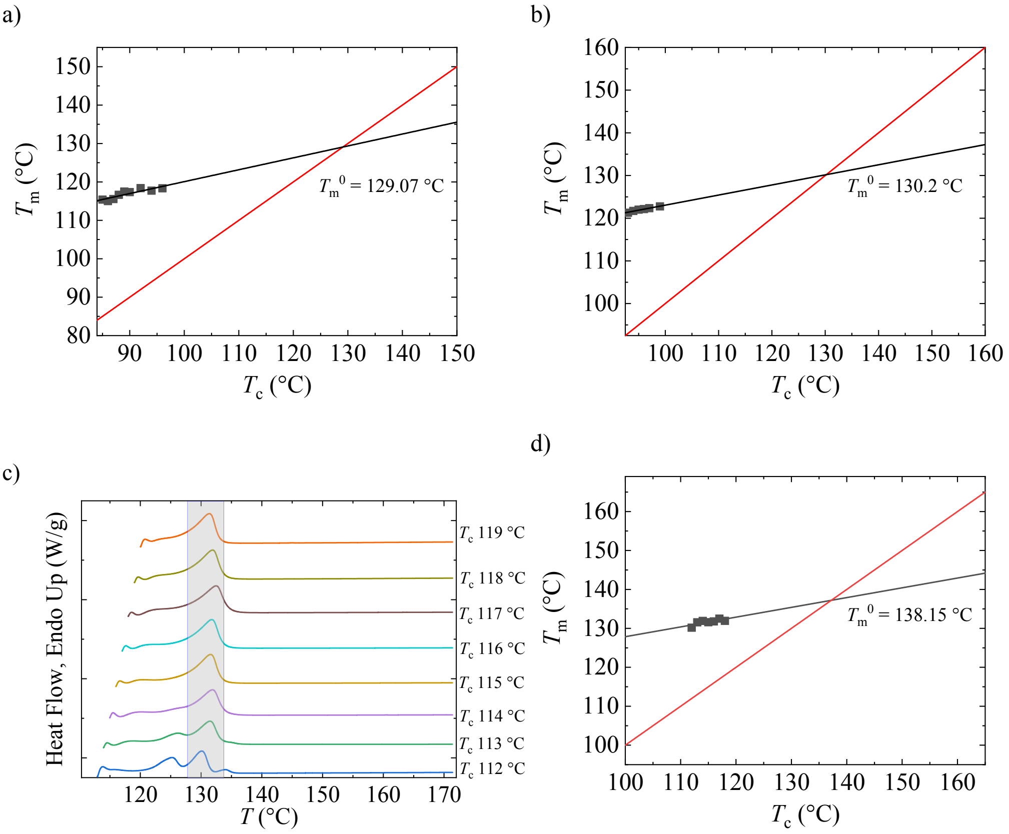

Figure S3. Melting temperature of the sample as a function of the crystallization temperature for a) PEA8-8, b) PEA8-10. c) Melting endotherms of PA8-16 crystallized at several T_c and d) T_m as a function of T_c.

Table S.1. ΔH is the melting enthalpy, M_i is the molar mass of the chemical structure and ΔH_m^0 the equilibrium melting enthalpy of the samples studied calculated from Van Krevelen group contribution theory.

Sample	Total n CH_2	ΔH (kJ/mol)	M_i (g/mol)	ΔH_m^0 (J/g)
PEA8-6	14	22	370	148.6
PEA8-7	15	59	384	153.6
PEA8-8	16	63	398	158.3
PEA8-9	17	67	412	162.6
PEA8-10	18	71	426	166.7
PEA8-11	19	75	440	170.5
PEA8-12	20	79	454	174.0
PEA8-13	21	83	468	177.4
PEA8-14	22	87	482	180.5
PEA8-15	23	91	496	183.5
PEA8-16	24	95	510	186.3
PEA8-17	25	99	524	188.9
PEA8-18	26	103	538	191.4
PEA8-19	27	107	552	193.8

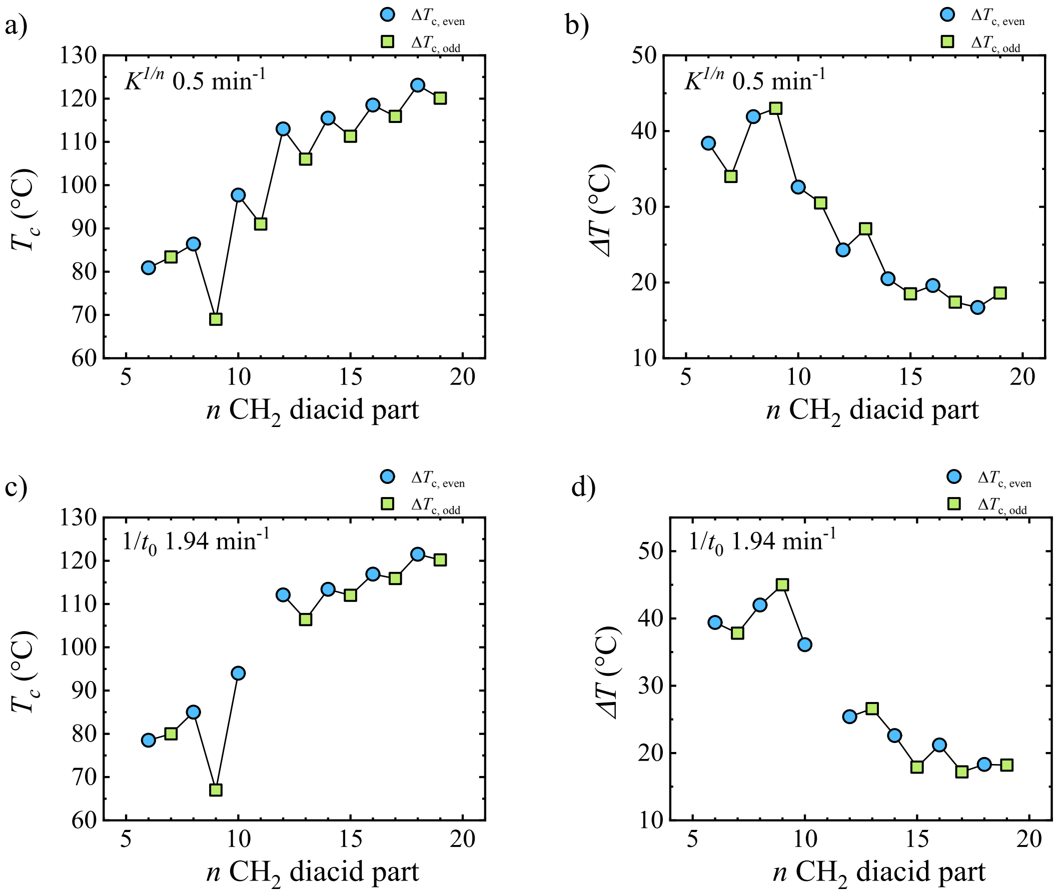

Figure S4. a) Crystallization temperature and b) supercooling needed to reach an overall crystallization rate constant equal to 0.5 min^{-1}. c) Crystallization temperature and d) supercooling needed to reach a value of inverse of the induction time equal to 1.94 min^{-1}.

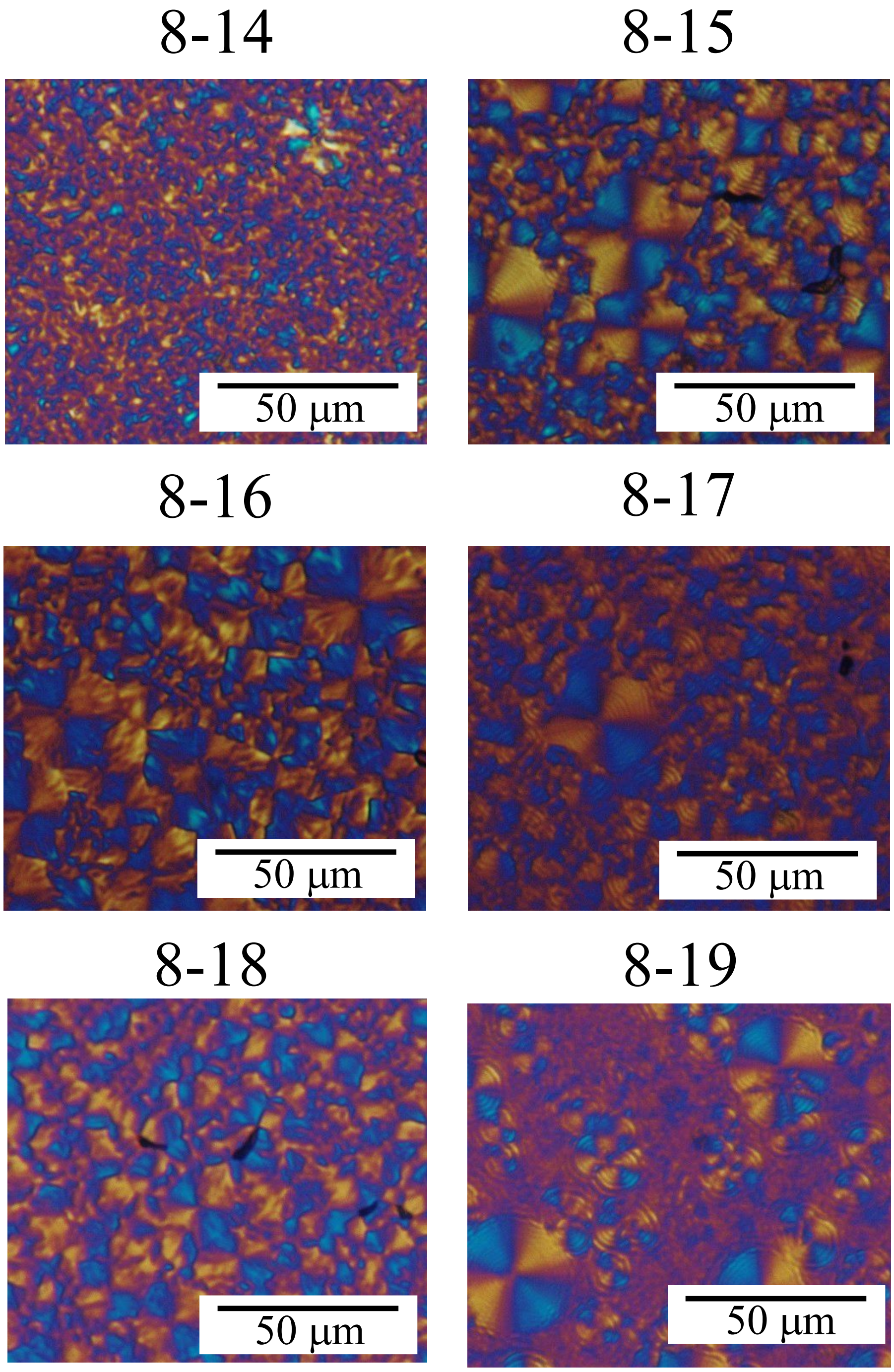

Figure S5. Magnification of the PLOM pictures acquired during non-isothermal experiments for PEAs with long alkyl chain length (PEA8-14 to PEA8-19).

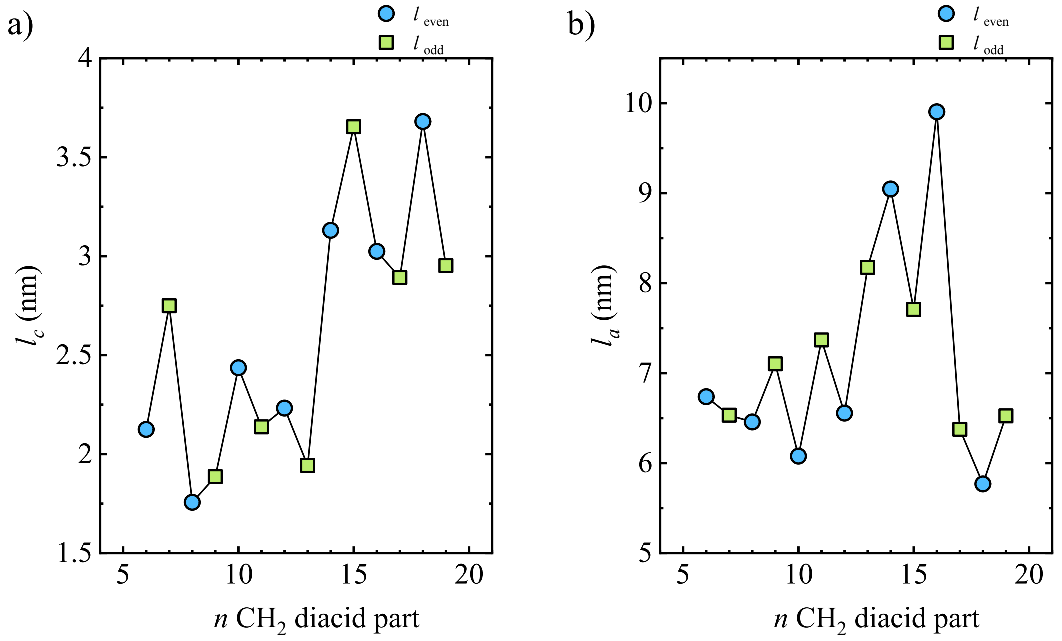

Figure S6. a) Crystalline lamellar thickness and b) amorphous layer thickness as a function of the number of CH_2 groups in the diacid part considering the crystallinity degree obtained by DSC, $l_c = X_c \times d$.

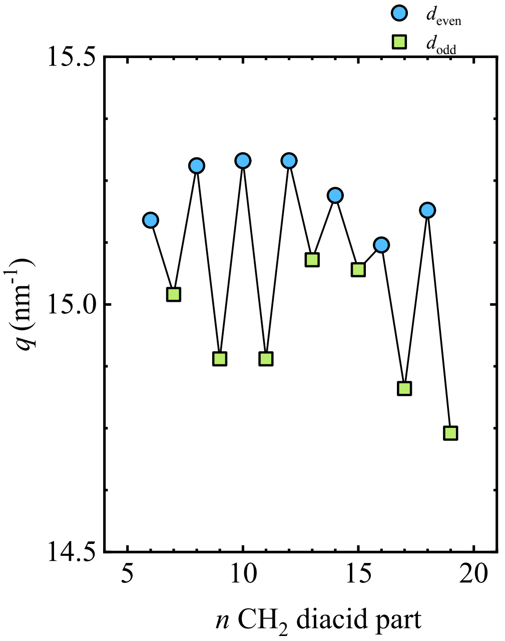

Figure S7. q values obtained from the main reflection as a function of the number of CH_2 groups in the diacid part.

References

1. Fernández-Tena, A., Pérez-Camargo, R. A., Coulembier, O., Sangroniz, L., Aranburu, N., Guerrica-Echevarria, G., Liu, G., Wang, D., Cavallo, D. Müller, A. J. Effect of Molecular Weight on the Crystallization and Melt Memory of Poly (ε-caprolactone)(PCL). Macromolecules 2023, 56, 4602-4620. https://doi.org/10.1021/acs.macromol.3c00234
